# Psychological outcomes and quality of life among first married women in polygynous families in Gedeo Zone, Southern Ethiopia: A mixed-approach study, 2021

**DOI:** 10.1371/journal.pmen.0000640

**Published:** 2026-06-15

**Authors:** Seid Shumye, Eden G/egziabher, Yesuneh Bayissa, Nebiyu Mengistu

**Affiliations:** 1 Department of Psychiatry, College of Health and Medical Science, Dilla University, Dilla, Ethiopia; 2 Department of Sociology, College of Social Science and Humanities, Dilla University, Dilla, Ethiopia; 3 School Medicine, Dilla University, Dilla, Ethiopia; PLOS: Public Library of Science, UNITED KINGDOM OF GREAT BRITAIN AND NORTHERN IRELAND

## Abstract

Polygynous marriage remains a culturally and socially embedded institution in many parts of Ethiopia, including the Gedeo Zone, where it is sustained by tradition religious belief, and community expectations. Despite its social acceptance, increasing evidence indicates that women in such unions experience elevated psychological distress and reduced quality of life, with first- married women facing particularly pronounced emotional and social challenges. This study aimed to assess the psychological outcomes and quality of life among first married women in polygynous families in the Gedeo Zone, Southern Ethiopia, 2021.A concurrent mixed-method design was employed, combining a cross-sectional survey of 381 first married women selected through multistage sampling with 10 in depth interviews and 4 focus group discussions. Standardized instruments such as the Depression, Anxiety, and Stress Scale (DASS‑21), the Rosenberg Self‑Esteem Scale (RSES), and the WHO Quality of Life‑BREF (WHOQOL‑BREF). The quantitative data were analysed using descriptive and inferential statistical analysis aided by SPSS version 25 program. Qualitative data were transcribed verbatim and analysed thematically.Moderate to severe depressive symptoms were reported by 48.6% participants, anxiety by 55.4%, and high stress levels by 41.2%. The mean (±SD) overall quality of life score was 52.3 ± 11.6, which falls below established norms and reflects generally poor life satisfaction. Lower educational status, socio‑economic dependency, lack of emotional spousal support, and presence of inter‑spousal conflict were significantly associated with higher psychological distress (p < 0.05). Thematic analysis revealed feelings of rejection, injustice, and emotional deprivation as dominant narratives among participants. Social approval of polygyny and religious sanction were commonly cited as reasons for acceptance of the practice despite its negative emotional outcomes.First married women in polygynous families in the Gedeo Zone experience marked psychological distress and reduced quality of life, calling for integrating psycho-social support, women’s empowerment, and marital counselling interventions.

## Background

Family systems constitute foundational elements of societal structure and fundamentally shape mental health outcomes across cultures. Polygyny defined as one husband with multiple wives represents the predominant form of plural marriage globally [1]. This practice spans approximately 850 societies worldwide [2]. It is accepted by a wide range of non-western ethnic and religious groups [3].

Historically, polygyny offered adaptive benefits in high-mortality contexts, including expanded household labour, lineage preservation through multiple offspring, and social prestige, particularly where child mortality exceeded 40% or female-to-male ratios favored plural marriage [4–7]. In many regions, polygyny served as a mechanism for economic resilience through diversified labour pools and kinship risk-sharing [8–10].

Sub-Saharan African polygyny faces growing scrutiny amid women’s rights movements [11–13]. Recent Demographic and Health Surveys document declining prevalence driven by urbanization, escalating bride wealth costs, rising female education levels, and shifting gender norms [14]. While some intellectuals appreciate the decline in the practice of polygamy, others are much dissatisfied with the trend [15,16]. This decline sparks debate among scholars, with some celebrating expanded women’s autonomy while traditionalists lament erosion of cultural heritage [17].

Ethiopia polygyny persists nationwide despite legal prohibition under Article 11 of the Revised Family Code (2000) [18]. The Ethiopian Demographic and Health Survey (EDHS) reports11% of women aged 15–49 in bigamous unions (8.2% with one co-wife, 2.6% with two or more) [19]. Prevalence remains stable from 2016 levels at 4.7% among men, peaking in pastoralist (Afar, Somali: 21–25%) and Muslim-majority regions [20–22].

Women in polygynous households consistently report dissatisfaction, families, particularly first wives experiencing acute distress when husbands introduce additional spouses [23]. These unions exhibit elevated rates of inter-spousal conflict, jealousy, and tension compared to monogamous relationships [24–29]. Polygynous family dynamics particularly predispose first wives and their children to mental health challenges [30].

Proponents cite economic benefits (shared agricultural labour), social prestige, and religious sanction, yet these rationales increasingly conflict with documented psychological costs [31,32]. Contemporary scholarship increasingly documents profound psychological tolls on women particularly first wives, who experience status displacement. Global studies consistently identify emotional deprivation, co-wife rivalry, polygyny-related chronic stress, and inequitable resource distribution as primary mechanisms [33,34]. Psychologically, first wives navigate complex emotional terrain, simultaneously internalizing cultural acceptance while grappling with jealousy, alienation, and diminished self-worth. Meta-analyses confirm significantly elevated depression (OR=2.31), anxiety (OR=2.12), and somatization rates among polygynous wives across Middle Eastern and African contexts [35–37]. Hierarchical household dynamics typically privilege newer wives, eroding first wives authority, social standing, and self-esteem [38].

Despite legal prohibitions polygyny thrives in rural, Muslim, and traditionalist communities [18,39]. Southern Ethiopia’s Gedeo Zone (1.3M population, 96% agrarian) exemplifies this persistence through syneretic religious-cultural reinforcement [19]. Religious endorsement (“blessings multiply”), economic mutualism, and patriarchal prestige sustain local prevalence at 14–19% triple urban averages [40]. This evidence gap impedes development of contextually appropriate mental health interventions for polygynous first wives.

Quality of life (QoL) among polygynous first wives can be framed through WHOQOL-BREF’s four domains: physical health (compromised nutrition/healthcare access), psychological well-being (chronic stress/self-esteem erosion), social relationships (co-wife rivalry/isolation), and environmental factors (economic dependency/autonomy loss) [41,42,37]. Resource inequity and emotional neglect precipitate role strain, diminished life satisfaction, reduced self-efficacy, and impaired social functioning [43]. First wives bear disproportionate emotional burdens post-remarriage: 65–72% reduced spousal attention, decision-making authority shifts to newer wives within 6–12 months, 94% cite co-wife rivalry as primary stressor [44].

Consequently, concurrent mixed-methods research integrating quantitative prevalence measurement with qualitative mechanism elucidation offers optimal approach for understanding these dynamics. Such evidence informs culturally grounded interventions promoting gender equity and psycho-social well-being within Ethiopia’s plural marriage contexts. Therefore, the aim of this study was assess psychological outcomes and quality of life among first married women in Gedeo Zone polygynous households, 2021.

## Methods

### Ethics statement

Institutional Review Board approval was secured from Dilla University Institutional Review Board (duirb/002/21–07), effective January 26, 2021 through January 25, 2022. Informed consent procedures included written documentation for literate participants and witnessed verbal consent for illiterate women, emphasizing voluntary participation, data confidentiality, and unrestricted withdrawal rights at any stage. Participants displaying acute psychologically distress received immediate intervention protocols: temporary interview suspension, empathetic support, and facilitated referrals to local health extension services with transportation assistance when needed.

### Study design and setting

Concurrent mixed‑method design was implemented during February-May 2021 with in Gedeo Zone, Southern Ethiopia (1.3 million population, 96% agrarian economy where polygyny persists at 14–19% prevalence due to entrenched cultural-religious norms).

### Population and sampling

Target population comprised all first wives living in polygynous households across three purposively selected districts (Yirga Cheffe, Bule, and Dilla Zuria). Multistage probability sampling employed: kebeles (Ethiopia’s smallest administrative units, typically serving 3,500–4,000 residents, analogous to neighbourhoods/wards) randomly selected (n = 12), from each district, followed by systematic households sampling using local administrative registries. Quantitative sample size calculated using single population formula, assuming 50% psychological distress prevalence (since there is no previously published articles regarding this issue in the study area), 5% margin of error, 95% CI, and 10% non‑response adjustment; final sample size was n = 381. Qualitative component utilized purposive sampling of 10 in-depth interview participants and 4 focus group discussions (6–8 participants each, total n = 28)) from survey respondents, selected for maximum variation across age, socioeconomic status, parity, and time since co-wife introduction, guided by information power/saturation principles.

### Data‑collection instruments

Quantitative assessment utilized structured interviewer‑administered questionnaires comprising five domains. Socio‑demographic characteristics and marital history captured via researcher‑developed items. Psychological distress evaluated using Depression Anxiety Stress Scale‑21 (DASS‑21) (α = 0.91 overall; depression α = 0.82, anxiety α = 0.77, stress α = 0.79), validated 21‑item measure employing a 4‑point Likert scoring across three sub-scales [45]. Self‑esteem assessed via Rosenberg Self‑Esteem Scale (RSES(α = 0.85), 10-item standardized instrument [46]. Quality of life measured with World Health Organization Quality of Life Scale (WHOQOL‑BREF)(domain α = 0.70-0.89) [47], across physical, psychological, social, and environmental domains. Perceived social support evaluated by Oslo‑3 Social Support Scale (OSSS-3) (α = 0.75) [48].

Qualitative data collection employed semi‑structured interview and focus group guides developed through literature synthesis and pilot testing with local experts. Guides explored lived emotional experiences, coping mechanisms, sociocultural perceptions, and subjective meanings of polygynous marriage. Interviews were conducted in Amharic and Gedeo‑Offa by trained female data collectors and were audio-recorded with participant consent. Each in-depth interview lasted, on average, 45 minutes (range: 35–60 minutes). Each FGD lasted, on average, 90 minutes (range: 75–120 minutes).

### Data‑collection procedure

Data collectors completed two‑day training covering ethical protocols, confidentiality maintenance, trauma-informed interviewing and instrument administration. All instruments underwent forward-backward translation (English → Amharic/Gedeo’uffa → English) verifying linguistic equivalence and cultural appropriateness. Pilot testing conducted with 5% sample (n = 19) from adjacent non-study, confirming clarity, timing, and cultural sensitivity. Quantitative interviews administered in participants’ homes (husband absent) or neutral community venues ensuring privacy; qualitative sessions conducted exclusively in community centres. Field supervisors conducted daily completeness audits and data consistency checks.

### Data analysis

Quantitative data double coded and entered into Epi-Data 3.1 and subsequently exported to SPSS version 25 for analysis. Descriptive statistics summarized socio‑demographics and scale domain means/standard deviations. Bivariate analyses employed independent t‑tests, one-way ANOVA, and chi‑square tests screening associations (p < 0.25 advancement criterion). Multivariable linear regression identified independent predictors (entry p < 0.25, retention p < 0.05) of psychological distress and quality-of-life outcomes. Statistical significance declared at α = 0.05 (95% confidence level).

### Qualitative rigor enhancement

Qualitative data underwent verbatim transcription, English translation, and N Vivo 12 import for thematic analysis following Braun & Clarke’s (2006) six-phase framework. Analysis phases: (1) data familiarization, (2) generating initial codes, (3) searching for themes, (4) reviewing themes, (5) defining/naming themes, (6) producing the report. Trustworthiness ensured through: credibility (Triangulation (IDI + FGD+ quantitative findings), prolonged engagement, member checking), dependability (audit trail (NVivo logs), code-recode), confirmability (reflexivity statement, peer debriefing), transferability (thick description). Key themes were triangulated with quantitative findings to strengthen contextual explanation.

### Data‑quality assurance

Several strategies were used to maintain data quality. Instruments underwent translation and back‑translation to ensure semantic accuracy. A pilot test helped refine ambiguous questions. Supervisors performed routine cross‑checks of questionnaires, and double data entry minimized errors. Reliability coefficients (Cronbach’s α) were computed for major psychometric scales. Internal consistency: all scales α ≥ 0.70 (DASS-21: 0.91; RSES: 0.85; WHOQOL-BREF: 0.70-0.89; OSSS-3: 0.75).

## Results

### Socio‑demographic characteristics

The socio‑demographic profile of 381 respondents reveals that nearly half (45.9%) were aged 30–39 years, and (64.3%) reported no formal education. The majority engaged primarily in domestic roles (64.8% identified as housewives), with 42.5% earning 1000–2000 ETB monthly. Most respondents resided in polygynous households with two or more co‑wives (40.4% with two wives, 31.5% with three or more), highlighting the multiplicity of co-wives as a key structure feature shaping psycho-social dynamics (Table 1).‌‌

**Table 1:** Socio‑Demographic Characteristics of First Married Women in polygynous Families, Gedeo Zone, 2021.

Variable	Category	Frequency (n)	Percent (%)
Age (years)	20–29	46	12.1
	30–39	175	45.9
	40–49	108	28.3
	≥ 50	52	13.7
Educational status	No formal education	245	64.3
	Primary (1–8)	78	20.5
	Secondary & above	58	15.2
Occupation	Housewife	247	64.8
	Small scale business	74	19.4
	Daily labor/farming	60	15.8
Monthly household income (ETB)	< 1000	143	37.5
	1000–2000	162	42.5
	> 2000	76	20.0
Number of co‑wives	One	107	28.1
	Two	154	40.4
	Three or more	120	31.5

### Psychological outcomes (depression, anxiety, stress)

Substantial proportions of respondents reported moderate‑to‑severe psychological distress: 48.6% for depression, 55.4% for anxiety, and 41.2% for stress. Average DASS-21 scores further indicate 48.7% elevated depression, 56.3% anxiety, and 41.4% stress, with no significant gender differences (p > 0.05). The overall psychological symptom burden was 41.2%, with 31.2% mild and 10.5% moderate–severe distress. Elevated scores were significantly associated with fewer household assets, perceived favoritism toward newer wives, and diminished social support (p < 0.05) (Table 2).

**Table 2:** Prevalence and Mean Scores of Psychological Outcomes (DASS‑21, n = 381).

Parameter	Mean ± SD	Mild (%)	Moderate (%)	Severe (%)	Total Moderate–Severe (%)
Depression	11.8 ± 6.1	18.6	30.4	18.2	48.6
Anxiety	12.5 ± 6.8	16.0	36.1	19.3	55.4
Stress	10.7 ± 5.3	21.0	28.8	12.4	41.2

### Quality of Life of first married women in polygynous families in Gedeo zone, Southern Ethiopia

WHOQOL‑BREF domain scores revealed that physical (58.1) and environmental (53.0) domains were above average, while psychological well-being (46.9) and social‑relationship (42.7) were below average. The overall mean quality‑of‑life score of 52.3 (SD ± 11.6) signifies reduced life satisfaction compare to normative Ethiopian urban data (60–65). No significant gender differences existed in overall QoL (p = 0.051), though younger women generally reported better QoL (Table 3).

**Table 3:** Mean Domain Scores of Quality of Life.

Domain	Possible Score Range	Mean ± SD	95% CI	Interpretation
Physical Health	0–100	58.1 ± 12.3	56.7–59.6	Fair
Psychological	0–100	46.9 ± 14.2	44.5–49.3	Poor
Social Relationships	0–100	42.7 ± 13.8	40.9–44.3	Low
Environment	0–100	53.0 ± 11.1	51.6–54.7	Moderate
Overall QOL	–	52.3 ± 11.6	50.6–53.8	Below average

### Association between Socio‑Demographic Variables and Psychological Outcomes (Distress)

See Table 4.‌‌

**Table 4:** Bivariate Analysis of Selected Factors Associated with Psychological Distress.

Variable	Category	Mean DASS Score ± SD	F/t Value	p‑Value
Educational status	No formal	37.4 ± 11.3	15.61	< 0.001
	Primary	31.2 ± 9.8		
	Secondary & above	27.6 ± 8.5		
Monthly income	< 1000 ETB	39.1 ± 10.5	12.27	< 0.01
	> 2000 ETB	28.3 ± 9.4		
Spousal support	Low	41.5 ± 10.8	16.22	< 0.001
	High	28.0 ± 8.7		

### Predictors of psychological distress (multivariable linear regression)

Multivariate linear regression determined that low educational attainment (β = 3.84), low household income (β = 2.97), lack of emotional spousal support (β = 4.61), and high marital conflict (β = 2.42) independently predicted greater psychological distress (all p < 0.01). The model explained 45% of the variation in psychological distress scores. (Adjusted R² = 0.45; F = 28.6; p < 0.001)), underscoring the interplay between socioeconomic status and relational dynamics (Table 5).

**Table 5:** Independent Predictors of Psychological Distress (n = 381).

Predictor Variable	β (Unstandardized)	Std. Error	Standard β	T	p‑Value
Low Education (ref = ≥ secondary)	3.84	0.87	0.36	4.43	< 0.001
Low Household Income (< 1000 ETB)	2.97	1.01	0.29	2.93	0.004
Lack of Emotional Spousal Support	4.61	0.92	0.45	5.01	< 0.001
Marital Conflict (High)	2.42	0.88	0.27	2.75	0.006
Constant	18.3	3.9	–	4.69	< 0.001

Model R² = 0.47; Adjusted R² = 0.45; F = 28.6, p < 0.001.

### Correlation between quality of life and psychological distress

Pearson correlation analyses revealed significant negative correlations between all WHOQOL-BREF domains and psychological‑distress indicators. The psychological domain(r = –0.59, p < 0.001) demonstrated the strongest inverse association, while physical (r = –0.38), social (r = –0.44), and environmental (r = –0.33) domains also showed statistically significant negative relationships (all p < 0.01). These findings confirm the interdependence between emotional well-being and overall quality of life (Table 6).

**Table 6:** Pearson Correlation Coefficients between WHOQOL‑BREF Domains and DASS‑21 Scores.

Variables	Depression	Anxiety	Stress	Overall DASS
Physical QOL	–0.38***	–0.32**	–0.29**	–0.41***
Psychological QOL	–0.56***	–0.48***	–0.43***	–0.59***
Social QOL	–0.44***	–0.39***	–0.30**	–0.46***
Environmental QOL	–0.33**	–0.27**	–0.21*	–0.34**

(*p < 0.05; **p < 0.01; **p < 0.001).

### Qualitative findings

Ten in‑depth interviews and 4 FGDs yielded five key themes reflecting first-wife perspective. (see Table 7).

**Table 7:** Summary of Qualitative Themes and Representative Quotes.

Theme	Illustrative Participant Quote	Interpretation
Emotional neglect and jealousy	“When he married the second wife, he stopped visiting my room often. I feel like a servant, not a wife.”	Persistent emotional deprivation impacts self‑worth.
Role conflict and competition	“We compete even for his attention during meals; it brings sadness.”	Continuous interpersonal tension generates stress and anxiety.
Social conformity and fear of stigma	“Our elders say accepting co‑wives is faith; rejecting means sin.”	Cultural compliance overrides personal suffering.
Coping through spirituality	“I pray every morning and cry to God; that calms my mind.”	Religious and spiritual coping serve as emotional buffers.
Desire for empowerment	“If I had my own income, I could decide without fear.”	Economic independence perceived as pathway to empowerment.

### Integration of quantitative and qualitative results

Joint interpretation of findings reveals strong convergence: Quantitative data confirmed high psychological distress (48–55%) and low QoL domains (≤ 55%), Qualitative themes explained social norms, inequitable resource allocation, and limited spousal attention driving these outcomes, Spiritual coping emerged as the primary resilience mechanism, mitigating distress among some subgroups.

## Discussion

This concurrent mixed‑methods investigation extends scarce empirical evidence regarding polygyny’s psycho-social impacts in Ethiopia by concurrently quantifying psychological symptomatology and elucidating lived experiences among first wives. The high prevalence of depressive symptoms (48.6%), anxiety (55.4%), and stress (41.2%) aligns closely with recent studies from Nigeria(52.3%), Syria(50.2%), Pakistan (49.8%) and Jordan(49.1%), where polygynous wives reported substantially higher psychological symptom scores than monogamous counterparts [35,49,50,51]. Interactions among emotional rivalry, resource deprivation economic insecurity, and constrained decision making authority coverage to amplify chronic stress responses directly corroborating role‑strain and resource-deprivation frameworks advanced by family‑stress theorists [52].

Our results underscore the salience of emotional neglect and perceived inequality as central determinants of adverse mental‑health outcomes. Women who reported poor spousal communication and favoritism towards junior wives experienced significantly higher distress scores (β = 4.22, p < 0.001). This aligns with observations from Kenya and Tanzania, where unequal attention from the husband predicted depression and psychosomatic complaints [53,54]. The qualitative findings: capturing feelings of rejection and diminished marital identity suggest that psychological distress arises not merely from the marital structure itself, but from the subjective meaning attached to fairness and belonging within that structure.

Social‑exchange and equity theories provide an interpretive lens for this phenomenon: when individuals perceive that the ratio of effort to reward in a relationship is imbalanced, emotional dissonance and dissatisfaction emerge [55]. Within polygynous systems, first wives often feel their early commitment is insufficiently reciprocated once additional wives are introduced.

The mean quality‑of‑life score of 52.3 represents substantially reduced life satisfaction compared to Ethiopian urban women’s normative range (average ~60–65) reported in urban women studies group 2020 [56]. Multivariable regression identified education (β = -3.12) and economic autonomy(β = -2.87) as protective factors, consistent with global evidence that women’s financial independence reduces marital vulnerability and enhances self‑concept [57,58]. Limited control over household income and the absence of alternative livelihood opportunities confine first wives to structural dependency, which reinforces chronic psycho-social stress.

These findings resonate with the conservation of resources (COR) theory, which posits that stress arises when first wives perceive loss of key resources s—such as financial stability, social support, or self‑esteem [59]. Within COR framework, the economic and emotional asymmetry in polygynous homes constitutes a chronic resource loss environment.

An important observation emerging from the qualitative data is the cultural normalization of polygynous and its legitimization through religious discourse. Although many first wives expressed inner dissatisfaction, they also demonstrated cognitive acceptance of the practice as a divine or socially obligatory institution. This cognitive dissonance, where personal emotions conflict with existing value systems illustrates the complex intersection between culture and mental health. Comparable findings have been reported by [60], who noted that women often cope by reinterpreting suffering as a religious duty or trial, thereby mitigating overt resistance but not necessarily reducing underlying distress.

### Implications for policy and practice

The study’s implications extend beyond academic interest to practical interventions addressing first wife vulnerability. Community‑based psycho-social counselling, women’s self‑help groups, and economic empowerment initiatives (vocational training, microcredit access) can buffer polygynous impacts. Health professionals should receive training in identifying marital dynamics-related distress and providing culturally sensitive support. Community and religious leaders can facilitate dialogue about equitable marital relationships and shared decision‑making, reducing stigma attached to mental‑health care seeking. First wives often serve as cultural custodians influencing younger generations, making their mental well‑being crucial for family cohesion and child development [61–63].

### Strengths and limitations

The concurrent mixed‑methods design provided both numerical measurement and qualitative depth, enabling validation and contextual interpretation of the results. However, the cross‑sectional nature limits causal inference, and reliance on self‑reported data may introduce recall or social desirability biases. Limited generalizability exists beyond Gedeo’s polygynous context. Future longitudinal and comparative studies between monogamous and polygynous unions would deepen understanding of temporal changes and coping trajectories.

## Conclusion and recommendations

First wives in Gedeo polygynous households experience substantial psychological distress and diminished quality of life, shaped by social norms, economic dependency, and relational inequities. The intersection of cultural tradition and gendered expectations perpetuates psychological vulnerability, demanding multi‑sectoral strategies: psycho-social support through community counselling, economic empowerment via skills training/microfinance, and marital counselling interventions promoting communication and equity.

## Supporting information

S1 Appendix Facilitation Guide. (DOCX)

## Abbreviations

AOR: adjusted odds ratio
CI: confidence interval
DASS-21: depression, anxiety, and stress scale (21-item)
EDHS: Ethiopian demographic and health survey
LMICs: low- and middle-income countries
RSES: Rosenberg self esteem scale
SD: standard deviation
SPSS: statistical package for the social sciences
WHOQOL: World Health Organization quality of life scale
WHO: World Health Organization

## References

[1] Zeitzen MK. Polygamy: A cross-cultural analysis. Routledge; 2020.

[2] Sinai M, Peleg O. Marital interactions and experiences of women living in polygamy: An exploratory study. Int J Psychol. 2021;56(3):361–77. doi: 10.1002/ijop.12726 33174243

[3] Thobejane TD, Flora T. An exploration of polygamous marriages: A worldview. Mediterr J Soc Sci. 2014;5(27):1058–66.

[4] Anaduaka US. Effects of polygyny on child health and determinants of birth registration: the case of Nigerian children. 2019.

[5] Chukwudeh OS, Ojo FE. Social context of child survival strategies among mothers in polygynous marriages in Ibadan, Nigeria. 2018.

[6] Smith-Greenaway E, Trinitapoli J. Polygynous contexts, family structure, and infant mortality in sub-saharan Africa. Demography. 2014;51(2):341–66. doi: 10.1007/s13524-013-0262-9 24402794 PMC3974908

[7] Strassmann BI. Polygyny, Family Structure, and Child Mortality. Adaptation and Human Behavior. Routledge; 2017. p. 49–68.

[8] Dessy S, Tiberti L, Tiberti M, Zoundi D. Polygyny and farm households’ resilience to climate shocks. 2021.

[9] Dessy S, Tiberti L, Tiberti M, Zoundi D. Polygyny and Drought Resilience in Village Economies: Evidence from Rural Mali. World Bank Econ Rev. 2025. doi: 10.1093/wber/lhaf020

[10] Tapsoba A. Polygyny and the economic determinants of family formation outcomes in sub-saharan africa. 2022.

[11] Fenske J. African polygamy: Past and present. J Dev Econ. 2015;117:58–73. doi: 10.1016/j.jdeveco.2015.06.005

[12] Ogunyomi IO, Casper WJ. The work-family interface and polygamy in Africa: A demands-resources perspective. Afr J Manag. 2021;7(2):196–215. doi: 10.1080/23322373.2021.1911471

[13] Vision AD. Beyond the Clash. Imperial Sexism: Why Culture and Women’s Rights Don’t Clash. 2025. 9 p.

[14] Adeola O, Evans O, Ngare I. Gender equality, climate action, and technological innovation for sustainable development in Africa. Springer; 2024.

[15] Chae S, Agadjanian V. The Transformation of Polygyny in Sub-Saharan Africa. Popul Dev Rev. 2022;48(4):1125–62. doi: 10.1111/padr.12524 37662544 PMC10469552

[16] Garenne M. Marriage in Sub-Saharan Africa. The Routledge Handbook of African Demography. Routledge; 2021. p. 151–80. doi: 10.4324/9780429287213-12

[17] George AS, Mamedova S. Celebrating self-love: Women’s customized divorce rings as symbols of resilience and renewal. Partners Universal International Innovation Journal. 2024;2(5):59–81.

[18] Zeleke M, Mossisa B, Gessesse FM, Idris AK, Shiferaw A, Hailu K, et al. Ethiopian Journal of Human Rights_Vol_3. Ethiop J Hum Rights. 2018;3.

[19] Tesfay GH. Note on: the adverse effects of polygamy on the rights of women: a case study in Gedeo and Sidama Zones. Haramaya L Rev. 2017;6:91–110.

[20] Cincotta R, Smith S. What future for the western sahel? The region’s demography and its implications by, 2045. 2021.

[21] Gurmu E, Gibson MA. Are wives and daughters disadvantaged in polygynous households? A case study of the Arsi Oromo of Ethiopia. Evol Hum Behav. 2017;39(2):160–5.

[22] Tesfay GH. Note On: The adverse effects of polygamy on the rights of women: a case study in Gedeo and Sidama Zones. 2017.

[23] Naseer S, Malik F. A study on jealousy, marital satisfaction, and mental health comparing the first and second wife in Pakistani polygynous families. 2021.

[24] Adedini SA, Odimegwu C. Polygynous family system, neighbourhood contexts and under-five mortality in sub-Saharan Africa. Dev South Afr. 2017;34(6):704–20. doi: 10.1080/0376835x.2017.1310030

[25] Ahinkorah BO. Polygyny and intimate partner violence in sub-Saharan Africa: Evidence from 16 cross-sectional demographic and health surveys. SSM Popul Health. 2021;13:100729. doi: 10.1016/j.ssmph.2021.100729 33511263 PMC7815814

[26] Ebrahim NB, Atteraya MS. Polygyny and Intimate Partner Violence (IPV) Among Ethiopian Women. Glob Soc Welf. 2020;8(3):213–20. doi: 10.1007/s40609-020-00194-0

[27] Jansen NA, Agadjanian V. Polygyny and Intimate Partner Violence in Mozambique. J Fam Issues. 2020;41(3):338–58. doi: 10.1177/0192513x19876075 33518874 PMC7845931

[28] Lawson D, Gibson MA. Polygynous marriage and child health in sub-Saharan Africa: What is the evidence for harm? DemRes. 2018;39:177–208. doi: 10.4054/demres.2018.39.6

[29] Uggla C, Gurmu E, Gibson MA. Are wives and daughters disadvantaged in polygynous households? A case study of the Arsi Oromo of Ethiopia. Evol Hum Behav. 2018;39(2):160–5. doi: 10.1016/j.evolhumbehav.2017.11.003

[30] Mengistu N, Shumye S, Tesfaye TS, Haile S, Bayisa Y, Yimer S, et al. Stressful life experience of the first married women in polygamous families in Gedeo zone, South Ethiopia: a qualitative study, 2021. BMC Psychol. 2022;10(1):40. doi: 10.1186/s40359-022-00753-4 35193677 PMC8864848

[31] Kassaw C, Shumye S. The prevalence of suicidal behavior and its associated factors among wives with polygamy marriage living in Gedeo zone, southern Ethiopia, 2020. PLoS One. 2021;16(10):e0259029. doi: 10.1371/journal.pone.0259029 34695161 PMC8544854

[32] Tsala Dimbuene Z, Ahinkorah BO, Amugsi DA. Measuring the effects of community polygyny on intimate partner violence: a multilevel modeling using nationally representative cross-sectional data. Reprod Health. 2025;22(1):93. doi: 10.1186/s12978-025-02037-7 40437598 PMC12121072

[33] Al-Krenawi A. Polygamous marriages: An Arab-Islamic perspective. Couple relationships in a global context: Understanding love and intimacy across cultures. Springer; 2020. p. 193–205.

[34] Jankowiak W, Sudakov M, Wilreker BC. Co-Wife Conflict and Co-operation. Ethnology. 2005;44(1):81. doi: 10.2307/3773961

[35] Analyzing the effects of polygyny on first-wives among Pashtun community in Quetta, Pakistan. Ann Hum Soc Sci. 2024. ‌‌

[36] Barut A, Mohamud SA. The psychosexual and psycho-social impacts of polygamous marriages: a cross-sectional study among Somali women. BMC Womens Health. 2023;23(1):669. doi: 10.1186/s12905-023-02830-1 38093249 PMC10720057

[37] Shaiful Bahari I, Norhayati MN, Nik Hazlina NH, Mohamad Shahirul Aiman CAA, Nik Muhammad Arif NA. Psychological impact of polygamous marriage on women and children: a systematic review and meta-analysis. BMC Pregnancy Childbirth. 2021;21(1):823. doi: 10.1186/s12884-021-04301-7 34903212 PMC8667458

[38] Ogolsky BG, Whittaker AM, Monk JK. Power in Families. Power in Close Relationships. Cambridge University Press; 2019. p. 143–72.

[39] Mishebo S. Traditional practices that affect women in Gumuz community and efforts made in mitigating them in Dibate woreda, Benishangul Gumuz National Regional State. St. Mary’s University; 2015.

[40] Farooq-e-Azam, Iram Rubab, Salahuddin A, Ahmed Usman. Polygamy in Islam: Cultural Pressures and Religious Justifications in Pakistan. JITC. 2021;11(2). doi: 10.32350/jitc.112.13 ‌‌

[41] Boltz M, Chort I. The Risk of Polygamy and Wives’ Saving Behavior. World Bank Econ Rev. 2016;33(1):209–30. doi: 10.1093/wber/lhw054

[42] Nemati Faqir F, Azadi M, Talebian Sharif J. The effects of Islamic spiritual dignity therapy in promoting quality of life among women affected by divorce. Spiritual Clin Pract. 2025;12(2):195–204. doi: 10.1037/scp0000370

[43] Gage AJ, Thomas NJ. Women’s Work, Gender Roles, and Intimate Partner Violence in Nigeria. Arch Sex Behav. 2017;46(7):1923–38. doi: 10.1007/s10508-017-1023-4 28695296

[44] Zaatut A, Haj-Yahia MM. Beliefs about wife beating among Palestinian women from Israel: The effect of their endorsement of patriarchal ideology. Fem Psychol. 2016;26(4):405–25. doi: 10.1177/0959353516647071

[45] Oei TPS, Sawang S, Goh YW, Mukhtar F. Using the Depression Anxiety Stress Scale 21 (DASS-21) across cultures. Int J Psychol. 2013;48(6):1018–29. doi: 10.1080/00207594.2012.755535 23425257

[46] Rosenberg M. Rosenberg self-esteem scale (RSE). Acceptance and commitment therapy. Measures package. 1965;61(52):18.

[47] Organization WH. The world health organization quality of life (WHOQOL)-BREF. The World Health Organization quality of life (WHOQOL)-BREF. 2004.

[48] Kocalevent R-D, Berg L, Beutel ME, Hinz A, Zenger M, Härter M, et al. Social support in the general population: standardization of the Oslo social support scale (OSSS-3). BMC Psychol. 2018;6(1):31. doi: 10.1186/s40359-018-0249-9 30016997 PMC6050647

[49] Adamu H, Oche OM, Abel IR, Garba KA, Zubairu BB. Prevalence and pattern of depression among women of polygamous and monogamous family settings in rural areas of Sokoto State, Nigeria. Cent Afr J Public Health. 2021;7(1):11–22.

[50] Al-Krenawi A, Kanat-Maymon Y. Psychological symptomatology, self-esteem and life satisfactions of women from polygamous and monogamous marriages in Syria. Int Soc Work. 2016;60(1):196–207. doi: 10.1177/0020872814562478

[51] Al‐Krenawi A. The Syrian refugees in Jordan. EC Psychol Psych. 2019;8:382–8.

[52] Pearlin LI, Lieberman MA, Menaghan EG, Mullan JT. The stress process. J Health Soc Behav. 1981;22(4):337–56. doi: 10.2307/2136676 7320473

[53] Ashuma BS. Factors That Sustain the Institution of Polygyny and Their Effects on Family Well-being Among the Wanga People of Western Kenya. University of Nairobi; 2019.

[54] Keefe S. Looking outside the marriage: Polygyny, infidelity, and divorce in coastal Tanzania. Gendered perspectives on international development: Working papers. 2019. 0_1-20 p.

[55] Cropanzano R, Anthony EL, Daniels SR, Hall AV. Social Exchange Theory: A Critical Review with Theoretical Remedies. Acad Manag Ann. 2017;11(1):479–516. doi: 10.5465/annals.2015.0099

[56] Mamuye N, Gotu B. Statistical analysis of urban quality of life (case study: Hawassa town, SNNP region, Ethiopia). Am J Theoret Appl Stat. 2015;4(6):547–54.

[57] Gonçalves VN, Ponchio MC, Basílio RG. Women’s financial well‐being: A systematic literature review and directions for future research. Int J Consumer Studies. 2021;45(4):824–43. doi: 10.1111/ijcs.12673

[58] Mboli MV, Jude FA. Financial literacy and women’s empowerment: The impact on family size and economic independence. ‌‌

[59] Hobfoll SE. Conservation of resource caravans and engaged settings. J Occupat Organ Psyc. 2011;84(1):116–22. doi: 10.1111/j.2044-8325.2010.02016.x

[60] Zangeneh M, Al-Krenawi A. Culture, diversity and mental health-enhancing clinical practice. Springer; 2019.

[61] Amato PR. The impact of family formation change on the cognitive, social, and emotional well-being of the next generation. Future Child. 2005;15(2):75–96. doi: 10.1353/foc.2005.0012 16158731

[62] Galvin KM, Braithwaite DO, Bylund CL. Family communication: Cohesion and change. Routledge; 2015.

[63] Al-Krenawi A, Slonim-Nevo V. The Psychosocial Profile of Bedouin Arab Women Living in Polygamous and Monogamous Marriages. Fam Soc J Contemp Soc Serv. 2008;89(1):139–49. doi: 10.1606/1044-3894.3718

