# Supplementary material for: Psychological outcomes and quality of life among first married women in polygynous families in Gedeo Zone, Southern Ethiopia: A mixed-approach study, 2021
Source: PLOS Ment Health. 2026 Jun 15;3(6):e0000640. doi: 10.1371/journal.pmen.0000640 (PMC13268168; doi:10.1371/journal.pmen.0000640)
Supplement: S1 Appendix — (DOCX) [file pmen.0000640.s001.docx]

**S1 Appendix: A Facilitation guide**

**IDI (semi-structured) – key questions**

1. Marriage history; when/how polygamy began.
2. Changes after the new wife arrived (time, money, respect, roles).
3. Emotional effects (sadness, worry, anger, jealousy); stress triggers.
4. Relationship with husband (support, conflict, communication).
5. Relationship with co-wife/wives (cooperation/conflict).
6. Quality of life: health, mood, social life, finances/safety.
7. Coping (religion, social support, income activities, avoidance).
8. Needed support/interventions (counselling, mediation, and empowerment).

**FGD – key prompts**

1. Community views and reasons for polygamy.
2. Common challenges for first wives.
3. Typical conflict types and resolution mechanisms.
4. Coping/support sources in the community.
5. Acceptable solutions (psychosocial support, economic support, counselling).
