# Supplementary material for: Psychological outcomes and quality of life among first married women in polygynous families in Gedeo Zone, Southern Ethiopia: A mixed-approach study, 2021
Source: PLOS Ment Health. 2026 Jun 15;3(6):e0000640. doi: 10.1371/journal.pmen.0000640 (PMC13268168; doi:10.1371/journal.pmen.0000640)
Supplement: Attachment — Submitted filename: Response to the Editor and Reviewers.pdf [file pmen.0000640.s003.pdf]

Response to the Editor and Reviewers

Dear Editor,

We sincerely thank you and the reviewers for the careful reading of our manuscript entitled "Psychological Outcomes and Quality of Life among First Married Women in Polygamous Families in Gedeo Zone, Southern Ethiopia: A Mixed-Approach Study, 2021." We appreciate the constructive comments, which have helped us improve clarity, methodological transparency, and the overall scholarly contribution of the paper.

We have revised the manuscript accordingly. Below we provide a point-by-point response, indicating the changes made and where they appear in the revised manuscript. W included the copy of the facilitation guide in the manuscript as Appendix.

Kind regards,

Seid Shumye Mulat (Corresponding Author)

On behalf of all authors

Reviewer #1

Comment 1 (Title)

The title is relevant and appropriate.

Response: Thank you. No change was made except changes the word polygamous”” into “”polygynous.

Comment 2 (Abstract)

The flow is systematic and appropriate.

Response: Thank you. Minor language edits were made for conciseness without changing content in the revised manuscript.

Comment 3 (Background: funnel approach)

You mention polygamy is practiced in 850 societies globally, but then discuss only Ethiopia. Please use a funnel approach: global → Africa → Ethiopia → Gedeo.

Response: We agree. We revised the Background to adopt a clear funnel structure: (A). global overview of polygamy/polygyny and associated mental health evidence, (B.) sub-Saharan Africa, (C). Ethiopia, and (D). study context (Gedeo Zone). We added relevant global and African literature to support this structure in the revised manuscript.

Comment 4 (Methods: define kebeles)

Please explain what “kebeles” are.

Response: We revised the Methods to define kebele as the smallest administrative unit in Ethiopia (similar to a neighbourhood/ward) and added this in brackets at first mention Methods—Population and Sampling.

Comment 5 (Results and Discussion)

Good attempt.

Response: Thank you. We refined a few transitions and strengthened interpretation where needed (Results/Discussion)

Comment 6 (Further research)

Suggest areas for further research.

Response: We added a "Recommendations for further research" paragraph proposing: longitudinal designs, comparative studies (monogamous vs polygamous; first vs later wives), intervention studies (economic empowerment/psychosocial support), and deeper qualitative work on coping, resilience, and service access.

Comment 7 (References: APA 7)

Adhere to APA 7th edition.

Response: We revised the reference list and in-text citations to follow APA 7th edition, including capitalization, italics, journal formatting, and DOI presentation where available (References section).

Comment 8 (Verdict)

Accept with minor changes.

Response: Thank you for the positive assessment.

Reviewer #2

Comment 1–2 (General/quantitative strengths)

The study addresses an important issue; quantitative section is well-written with appropriate methods and consistent results.

Response: We appreciate this positive feedback.

Comment 3 (Qualitative component limited; lacks trustworthiness)

Qualitative section is limited in scope and does not meet qualitative standards (trustworthiness).

Response: We agree that the qualitative component required clearer reporting of rigor. In the revised manuscript, we strengthened the qualitative methodology by explicitly describing trustworthiness strategies (credibility, dependability, confirmability, transferability), including:

- ✓ Training and supervision of interviewers,
- ✓ Use of audio-recording and verbatim transcription,
- ✓ Iterative coding and team discussion of codes/themes,
- ✓ Triangulation across IDIs and FGDs and with quantitative findings, and
- ✓ Inclusion of illustrative quotes linked to themes.

We also clarified the purpose of the qualitative component as contextual explanation (why distress and poor QoL occur; coping and social norms) rather than statistical generalization.

Comment 4 (Recommendation: remove qualitative and keep quantitative only)

Recommend focusing exclusively on quantitative component.

Response: We respectfully disagree, and we request to retain the mixed-methods design. Our rationale is

- ✓ The quantitative findings demonstrate prevalence and predictors, but do not explain mechanisms (e.g., perceived injustice, emotional deprivation, stigma/fear of disclosure).
- ✓ The qualitative findings add interpretive depth and help ensure that proposed recommendations are culturally grounded.
- ✓ Mixed-methods is increasingly recommended for complex social/mental health phenomena in LMIC contexts.

However, we have tightened the qualitative component to ensure it meaningfully contributes, and we have improved reporting transparency and trustworthiness as described above.

Reviewer #3

We thank the reviewer for the detailed and constructive suggestions. We have revised the introduction, strengthened the quality-of-life conceptual framing, updated literature, and expanded methodological detail.

Comment 1 (Terminology: polygamy vs polygyny)

Polygamy is general; polygyny is more appropriate. If using polygamy, define and justify.

Response: We agree. We revised the introduction to define polygamy (multiple spouses) and clarify that the study context involves polygyny (one man, multiple wives). We now use "polygyny" where technically appropriate while retaining "polygamy" only when referring to broader literature or common usage in local policy discourse

Comment 2 (Economic explanations + outdated citations; add recent study)

Old studies; add recent literature e.g., Boltz & Chort (2019).

Response: We updated the economic rationale section and added newer literature, including Boltz & Chort (2019) on polygamy risk and wives' saving behavior, and other recent studies where relevant.

Comment 3 (Explain EDHS)

Explain first, then put EDHS in brackets.

Response: Done. We now write "Ethiopian Demographic and Health Survey (EDHS)" at first mention

Comment 4 (QoL concept underdeveloped; define QoL in polygamous marriage; justify first-wife focus)

Need deeper discussion of QoL; explain why first wife is key.

Response: We agree and have significantly revised this section by:

Expanding conceptualization of QoL using the WHOQOL domains (physical, psychological, social relationships, environment) and linking them to polygynous household dynamics (resource allocation, emotional support, social standing, safety, autonomy), acknowledging that QoL may be positively perceived in some contexts (e.g., shared labour/support) but emphasizing that inequity and conflict can reduce QoL, and adding a clearer rationale for focusing on first wives (status disruption after husband remarries, perceived loss of affection/resources, increased conflict/jealousy, and power reconfiguration within the household).

Comment 5 (Focus: socio-cultural vs QoL)

Socio-culture has nothing to do with QoL; clarify focus.

Response: We clarified that socio-cultural norms are not the primary outcome, but they are contextual determinants shaping psychological wellbeing and QoL (e.g., stigma, social conformity, religious legitimization). We revised wording to keep the manuscript centered on psychological outcomes and QoL, using socio-cultural factors only as explanatory context.

Comment 6 (Correct sentence: not psychological outcomes with socio-cultural factors)

Refine paragraph.

Response: We revised the paragraph to clearly state that we assessed psychological outcomes and QoL, and explored contextual factors qualitatively; we removed any over-claim that we quantitatively modeled "socio-cultural factors" as primary predictors.

Comment 7 (Ethics code; consent procedures; husband permission?)

Include ethics approval code and explain consent; did you need husband permission?

Response: We strengthened the Ethics and data-collection description by:

- ✓ Adding the ethical approval reference/approval number
- ✓ Expanding informed consent procedures (privacy, voluntary participation, confidentiality, right to withdraw), and
- ✓ Clarifying that participants provided their own informed consent. We did not require husbands' permission as a condition of participation; however, we arranged interviews in a way that ensured privacy and safety for participants (neutral locations/appropriate timing) and minimized risk of household conflict

Comment 8 (Qualitative sample determination theory)

Based on what? Saturation (Smith/Creswell)? Citation needed.

Response: We clarified that the qualitative sample size was guided by information power/saturation principles and practical feasibility, and we added supporting citations consistent with qualitative methodology guidance.

Comment 9 (Reliability of each quantitative tool)

Include reliability of each test.

Response: We added Cronbach's alpha values for each scale (DASS-21 subscales, RSES, WHOQOL-BREF domains, OSSS-3) calculated from our dataset (or, if not calculated, we reported validated Ethiopian/LMIC reliability where available and clarified accordingly). This is now included in data quality assurance section.

Comment 10 (Validation of interview guide; Cohen's Kappa; reflexivity)

Were questions validated? Kappa? reflexivity.

Response: We revised the qualitative methods section to state that the interview/FGD guide was reviewed by subject experts (mental health and sociology) and piloted. Regarding Cohen's Kappa, we clarify that Kappa is typically used for inter-rater agreement in structured coding;

in our study, we conducted team-based coding discussions and consensus building. We added a brief note on researcher reflexivity (positionality, training, minimizing bias)

Comment 11 (Handling sensitive issues during interviews; cultural alignment)

How manage distress; culturally sensitive questions?

Response: We added a description of distress and risk management procedures: pausing interviews, offering breaks/termination, providing supportive listening, referral pathways to nearby services, and using trained female data collectors. We also stated that questions were phrased respectfully in local languages, piloted for cultural appropriateness, and conducted in private settings.

Comment 12 (Subjective meaning)

Explain subjective meaning and interpretation.

Response: We revised the relevant sentence to define subjective meaning as participants' own interpretations of their experiences, emotions, and the personal significance of living in a polygynous marriage.

Comment 13 (Implications should be practical for first wife; avoid policy focus)

Focus on strategies to improve QoL and psychological wellbeing for first wife.

Response: We revised the Implications section to emphasize practical and individual-/family-level strategies, including:

- ✓ Psychosocial counseling and peer-support groups,
- ✓ Conflict resolution and communication support,
- ✓ Economic empowerment and skills training,
- ✓ Strengthening social support networks, and
- ✓ Referral and linkage to mental health services.

We reduced broad policy language and reframed recommendations to be more feasible and directly relevant to first wives and local service providers.
