# Supplementary material for: Psychological outcomes and quality of life among first married women in polygynous families in Gedeo Zone, Southern Ethiopia: A mixed-approach study, 2021
Source: PLOS Ment Health. 2026 Jun 15;3(6):e0000640. doi: 10.1371/journal.pmen.0000640 (PMC13268168; doi:10.1371/journal.pmen.0000640)
Supplement: Attachment — Submitted filename: Response to Reviewers.pdf [file pmen.0000640.s004.pdf]

Dear Editor and Reviewer,

We sincerely thank editor and reviewers for the careful reading of our manuscript and for the constructive and encouraging comments. We are pleased that the reviewers found the paper well written, interesting, and methodologically sound, and that most of the earlier suggestions were successfully addressed. We also appreciate the remaining suggestions, which helped us improve the clarity and precision of the manuscript further. Below, we respond point by point to the reviewer's comments.

Sincerely,

Seid Shumye(corresponding author)

On behalf of all authors

Reviewer#1 Comments

Comment#1: Abstract: The author indicates that quantitative data were analysed using SPSS version 25, applying descriptive statistics, bivariate and multivariable linear regression analyses. However, it would be best to indicate that quantitative data were analysed using descriptive and inferential statistical analysis aided by SPSS version 25 program. Since SPSS is not an analysis method but a tool for analysis.

Response: Thank you for this helpful comment. We have revised the abstract to state that the quantitative data were analysed using descriptive and inferential statistical analyses with the aid of SPSS version 25. This wording more accurately reflects SPSS as a statistical software tool rather than an analytical method.

Comment#2: Background of the study: This has been adequately addressed.

Response: We sincerely thank the reviewer for confirming that the background section has been adequately addressed.

Comment#3: Methodology: Well addressed as per the previous comments given.

Response: We appreciate the reviewer's positive assessment of the methodology section and are grateful that the previous concerns have been satisfactorily addressed.

Comment#4: Population and sampling: The author indicates that kebeles were randomly selected…. What or who are these kebeles. Write in brackets the meaning.

Response: Thank you for this important clarification. We have revised the manuscript to define kebeles at first mention as the smallest administrative units in Ethiopia, comparable to local neighbourhoods or communities, and have added this explanation in brackets for clarity.

Comment#5: Strengths and limitations: The author indicates that mixed methods design was used. However, which mixed methods design? There are six types of mixed methods research design, be specific on the exact MMR research design.

Response: We thank the reviewer for this important observation. We have now specified the exact mixed methods (concurrent or convergent parallel) research design used in the study and revised the manuscript accordingly to clearly identify the design and remove any ambiguity.

Comment#6: References: Align the references, some have CAPS others are indented while others are not.

Response: We appreciate this helpful comment. The reference list has been carefully revised to ensure consistency in capitalization, indentation, and formatting in line with the journal's requirements.

Reviewer#2 Comment

Reviewer #2: I find the paper well written and interesting. Quantitative methodology is adequate, and the findings are rooted in the literature. Most of my previous suggestions were corrected. However, I still think that it is advised to focus in this paper on the quantitative study and write another paper about the qualitative study. I leave this decision to the editor. In conclusion — accept for publication.

Response: We sincerely thank Reviewer #2 for the positive evaluation of our manuscript and for recognizing the clarity of the writing, the adequacy of the quantitative methodology, and the relevance of the findings to the literature. We also appreciate the reviewer's acknowledgment that most of the earlier concerns have been addressed. Regarding the suggestion to focus only on the quantitative component and prepare a separate paper for the qualitative findings, we respectfully note that this manuscript was intentionally designed as a concurrent mixed-methods study. The qualitative findings were included not as a separate strand, but as an essential complement to the quantitative results, helping to explain and contextualize the patterns observed in the survey data. In particular, the qualitative narratives provide important insight into the lived experiences, social norms, emotional neglect, and coping strategies of first married women in polygynous families, thereby strengthening the interpretation and practical relevance of the quantitative results. For this reason, we believe retaining both components in a single manuscript is appropriate and consistent with the study objectives. Nevertheless, we fully respect the reviewer's recommendation and will defer to the editor's judgment on the final presentation of the manuscript. We are grateful for the reviewer's overall support and recommendation for publication.
